# Supplementary material for: Coupling microwave photons to a mechanical resonator using quantum interference
Source: Nat Commun. 2019 Nov 25;10:5359. doi: 10.1038/s41467-019-12964-2 (PMC6877727; doi:10.1038/s41467-019-12964-2)
Supplement: Supplementary file 1 — Supplementary Information [file 41467_2019_12964_MOESM1_ESM.pdf]

Supplementary Material: Coupling microwave photons to a mechanical resonator using quantum interference

I. C. Rodrigues†, D. Bothner†, and G. A. Steele

Kavli Institute of Nanoscience, Delft University of Technology, PO Box 5046, 2600 GA Delft, The Netherlands
†these authors contributed equally

SUPPLEMENTARY NOTE 1: DEVICE FABRICATION

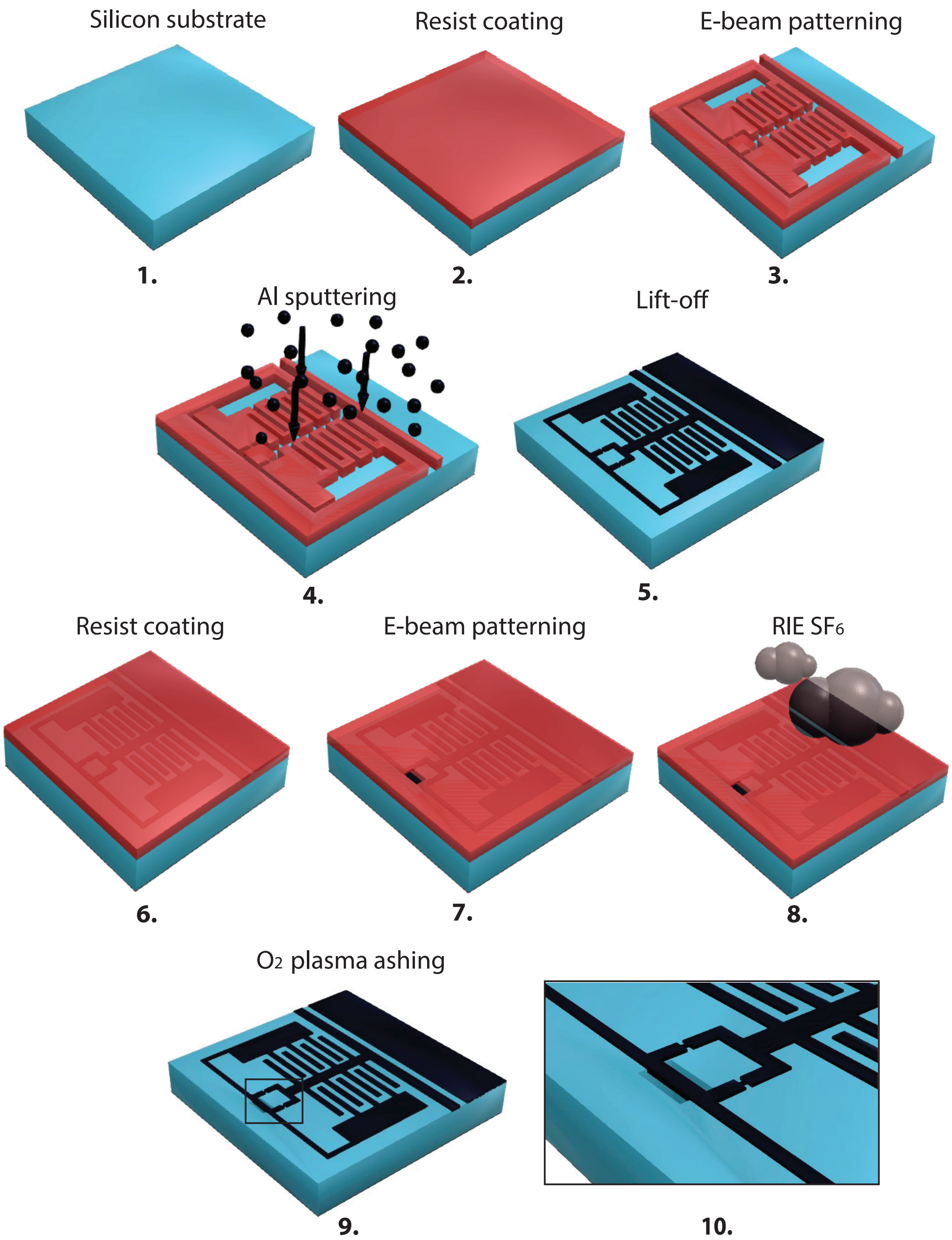

SUPPLEMENTARY FIGURE 1. **Schematic device fabrication. a 1.-5.** show the deposition and patterning of the superconducting microwave structures and **6.-9.** show the etching window patterning and nanobeam release. **10.** Zoom-in of a SQUID loop with a released beam. Dimensions are not to scale. A description of the individual steps is given in the text.

The fabrication of the device starts by patterning alignment markers (made of a 100 nm layer of sputter-deposited Molybdenum-Rhenium alloy) on top of a 2 inch silicon wafer. CSAR62.13 was used as patterning mask for the subsequent EBL (Electron Beam Lithography) step and warm Anisole at $\sim 80\,^{\circ}\mathrm{C}$ as solution for the lift-off process. Afterwards the wafer was diced in $14 \times 14\,\mathrm{mm}^2$ chips which were then individually used for the following fabrication steps.

The superconducting structures were patterned in a single EBL step where CSAR62.09 was used as resist. Posterior to the exposure, the sample was developed in Pentylacetate for 60 seconds followed by a solution of MIBK:IPA (1:1) for another 60 seconds and finally rinsed in IPA.

Once the mask was developed, the chip was loaded into a sputtering machine where a thin 20 nm layer of Aluminum (1% Silicon) was deposited after a short in-situ cleaning step by means of Argon ion milling. After the deposition, the sample was placed in the bottom of a beaker containing a small amount of room-temperature Anisole and left in a ultrasonic bath for a few minutes. During this time, the patterning resist is dissolved and the Aluminum layer sitting on top is lifted off.

At this point in the fabrication all the superconducting structures were patterned, leaving the most sensitive step, the mechanical release, for the end. Before the final release, however, the sample is once again diced to a smaller $10 \times 10\,\mathrm{mm}^2$ size in order to fit into the sample mountings and PCBs (Printed Circuit Boards).

For the final EBL step, a CSAR62.09 resist was once again used as mask and the development of the pattern was done in a similar way as for the first layer. Once the etch mask (consisting of two small windows enclosing one arm of the SQUID loop) was patterned, the sample underwent an isotropic SF_6 etch (at approx. ($\sim -10\,^{\circ}\mathrm{C}$) for two minutes. During this time the substrate under the beam is etched without attacking the thin aluminum layer forming the cavity and the mechanical beam. Once the beams are released, we proceeded with a O_2 plasma ashing step in order to remove the remaining resist from the sample.

In the end of the fabrication, the sample is glued to a PCB and wire-bonded both to ground and to the $50\,\Omega$ connector lines. A schematic representation of this fabrication process can be seen in Supplementary Fig. 1, omitting the patterning of the electron beam markers.

SUPPLEMENTARY NOTE 2: MEASUREMENT SETUP

All the experiments reported in this paper were performed in a dilution refrigerator operating at a base temperature $T_b \approx 15\,\mathrm{mK}$. A schematic of the experimental setup and of the external configurations used in the different experiments can be seen in Supplementary Fig. 2.

The PCB (Printed Circuit Board) onto which the fabricated sample was mounted, was placed in a radiation tight copper housing and connected to three high frequency coaxial lines. For a rudimentary shielding of magnetic out-of-plane noise without impacting significantly the in-plane magnetic field, a thin superconducting Aluminum cover was placed in parallel $\sim 1\,\mathrm{mm}$ above the chip (not shown in the schematic).

Two of the coaxial lines were used as standard input and output microwave lines, used to measure the SQUID cavities in a side-coupled transmission configuration. Furthermore, in order to generate an out-of-plane magnetic field component, required for the tuning of the SQUID, and for the Lorentz-force actuation of the mechanical resonator, DC currents and low-frequency (LF) signals were sent via a third input line. To combine the DC and the LF signals, the center conductor of the coaxial cable was connected to a DC wire by means of a bias-tee.

All coaxial input lines were heavily attenuated in order to balance the thermal radiation from the line to the base temperature of the refrigerator. Outside of the refrigerator, we used different configurations of microwave signal sources and high-frequency electronics for the three experiments. A representation of the setups can be seen in Supplementary Figs. 2**b**, **c** and **d**, where the setup for the thermomechanical noise detection is shown in **b**, the setup for the up-conversion of mechanical motion in **c**, and the setup for optomechanically induced transparency is shown in **d**. A detailed schematic of the connections inside the cooper housing box is shown in **e**, and the symbol legend is given in **e**.

For all experiments, the microwave sources and vector network analyzers (VNA) as well as the spectrum analyzer used a single reference clock of one of the devices.

Estimation of the attenuation chain

To estimate the microwave power on the on-chip microwave feedline, we follow two distinct approaches. First, we add all specified loss elements like attenuators or directional couplers. Then, we estimate the total additional losses induced by non-specified components like cables and connectors based on a transmission measurement and attribute those additional losses to input and to output cabling, giving significantly more weight to the input lines

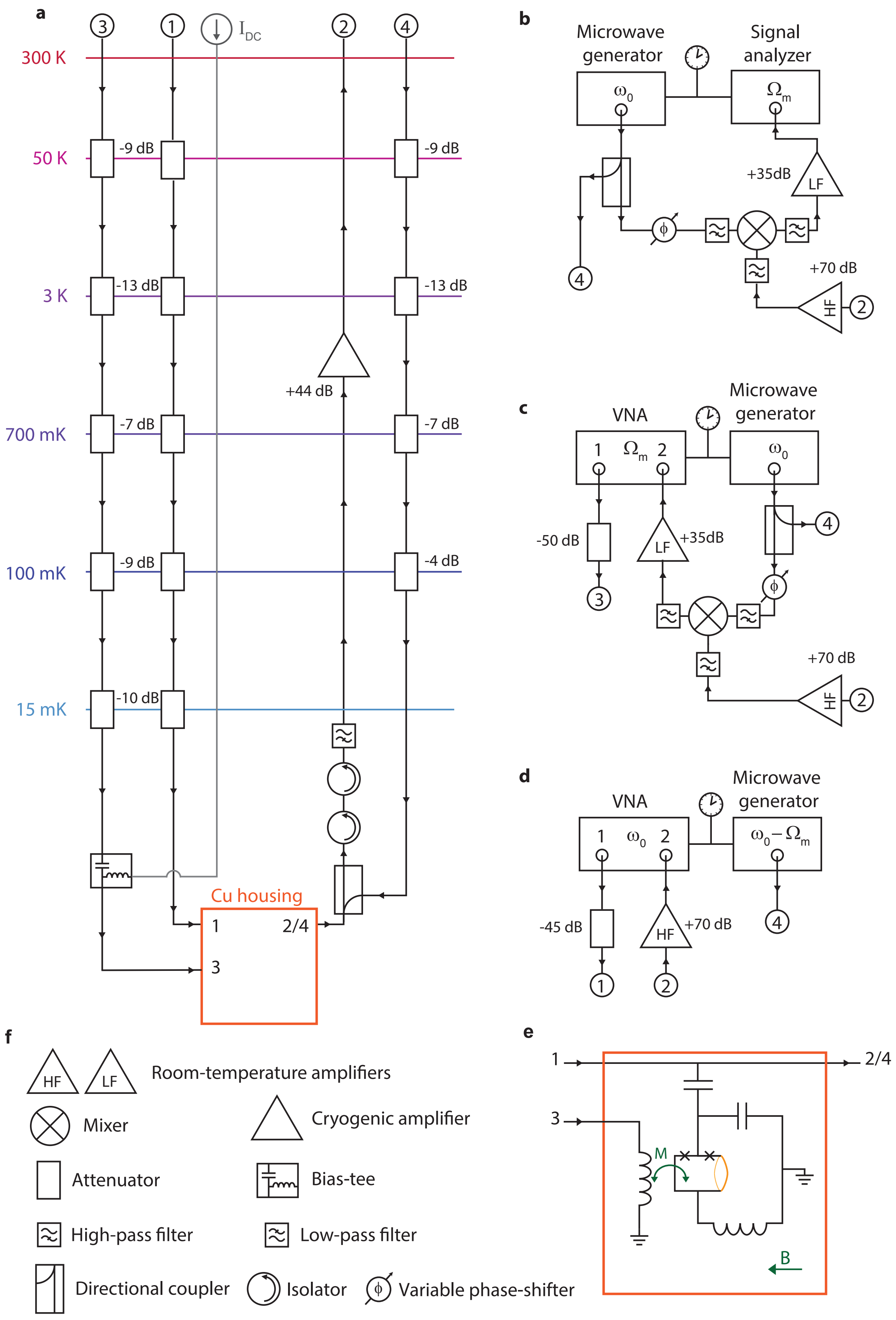

SUPPLEMENTARY FIGURE 2. **Schematic of the measurement setup.** Detailed information is provided in text.

due to the longer input cables with more potentially lossy connectors. For the probe signal line 1, we measure an average transmission of $-5\,\mathrm{dB}$, when having $45\,\mathrm{dB}$ room-temperature attenuation, $48\,\mathrm{dB}$ cryogenic attenuation, $44\,\mathrm{dB}$ cryogenic gain at the HEMT amplifier and $70\,\mathrm{dB}$ gain by room-temperature amplifiers. This leaves about $26\,\mathrm{dB}$ of unaccounted losses, of which we attribute about $17\,\mathrm{dB}$ to the input and $9\,\mathrm{dB}$ to the output line, respectively. In total, this corresponds to an input attenuation of $-110\,\mathrm{dB}$.

Assuming a similar procedure for the pump input line (line number 2), we get a total attenuation of $\sim -68\,\mathrm{dB}$ there.

As second approach, we consider the thermal noise of the HEMT amplifier as calibration standard. The HEMT noise power can be determined by

$$P_{\mathrm{HEMT}} = 10\log\left(\frac{k_{\mathrm{B}}T_{\mathrm{HEMT}}}{1\,\mathrm{mW}}\right) + 10\log\left(\frac{\Delta f}{\mathrm{Hz}}\right) \tag{1}$$

where k_{B} is the Boltzmann constant, T_{HEMT} is the HEMT noise temperature and according to the data sheet is $T_{\mathrm{HEMT}} \approx 2\,\mathrm{K}$. The measurement IF bandwidth of our calibration measurement is $\Delta f = 1\,\mathrm{kHz}$. In total, we get with these numbers $P_{\mathrm{HEMT}} = -165.6\,\mathrm{dBm}$ or the corresponding noise RMS voltage $\Delta V = 1.66\,\mathrm{nV}$. From the signal-to-noise ratio of SNR$=34.2\,\mathrm{dB}$ for a VNA output power of $-20\,\mathrm{dBm}$, we then get the signal power arriving at the HEMT input as $-131.4\,\mathrm{dBm}$. Assuming an attenuation between the sample and the HEMT of $2\,\mathrm{dB}$ leaves us with a total input attenuation between VNA output and sample of $-109.4\,\mathrm{dB}$.

When performing this procedure with the pump line, we get about $-66\,\mathrm{dB}$ of attenuation.

For the calibration of the photon numbers in this paper we therefore work with the attenuations $G_{\mathrm{signal}} = -110\,\mathrm{dB}$ and $G_{\mathrm{pump}} = -67\,\mathrm{dBm}$ in good agreement with both methods and estimate the accuracy of the achieved calibration on the order of $3\,\mathrm{dB}$. Note that in addition to the uncertainty mentioned here, the power arriving on the chip is also frequency dependent, as we usually observe background transmission oscillations of about 2 dB peak-to-peak amplitude due to cable resonances. Therefore, the experimental single-photon coupling-rate g_0, calculated by using an estimate of the on-chip power based on the attenuation chain, has an estimated uncertainty $\approx \pm 0.2 g_0$.

SUPPLEMENTARY NOTE 3: CAVITY CHARACTERIZATION

Cavity modelling

1. Interdigitated capacitors

The two interdigitated capacitors C of our device consist of $N = 120$ fingers each, with finger and gap widths of $1\,\mu\mathrm{m}$ and a finger length $l_f = 100\,\mu\mathrm{m}$. With the relative permittivity $\epsilon_r = 11.8$ of the Silicon substrate and using the equations given in Ref.[1] we calculate the capacitance of one of the main interdigitated capacitors to be $C = 680\,\mathrm{fF}$ and the interdigitated part of the coupling capacitor as $C_c' = 27\,\mathrm{fF}$.

For the total coupling capacitance, we also have to take into account the capacitance between the center conductor of the feedline and the fingers of both cavity capacitors C. We do this by calculating the feedline capacitance per unit length $C' = 144\,\mathrm{pF/m}$ and with a total length of $204\,\mu\mathrm{m}$ we get $29\,\mathrm{fF}$. The capacitance between the center conductor and the cavity center electrode, however, is only approximately a factor of 0.25 of that, such that $C_c = 34\,\mathrm{fF}$.

The resonance frequency of the circuit is $\omega_0 = 2\pi \cdot 5.221\,\mathrm{GHz}$ and related to the circuit parameters by

$$\omega_0 = \frac{1}{\sqrt{L_{\mathrm{tot}}(2C + C_c)}} \tag{2}$$

where the total inductance $L_{\mathrm{tot}} = (L + L_J)/2$. The linear inductance L is a combination of the SQUID loop inductance L_l and other linear inductance contributions in the circuit. All those have a geometric and a kinetic contribution and from the SONNET simulations discussed below, we estimate the kinetic contribution of the linear inductance to be $L_k = 0.73L$. The total inductance is approximately $L_{\mathrm{tot}} = 666\,\mathrm{pH}$. This value is in good agreement with numbers we got using numerical inductance calculation of the whole device and assuming a London penetration depth $\lambda_{\mathrm{L}} = 160\,\mathrm{nm}$, which corresponds to $L_k \approx 2.75 L_g$.

2. SONNET simulations and the kinetic inductance

We simulated the cavity with the software package SONNET to determine the kinetic inductance per square $L_\square$. For a vanishing surface impedance we find a resonance frequency $\omega_{00} = 2\pi \cdot 10.05\,\mathrm{GHz}$ and achieve high agreement with the experimental value of $\omega_0 = 2\pi \cdot 5.221\,\mathrm{GHz}$ when $L_\square = 2.3\,\mathrm{pH/sq}$.

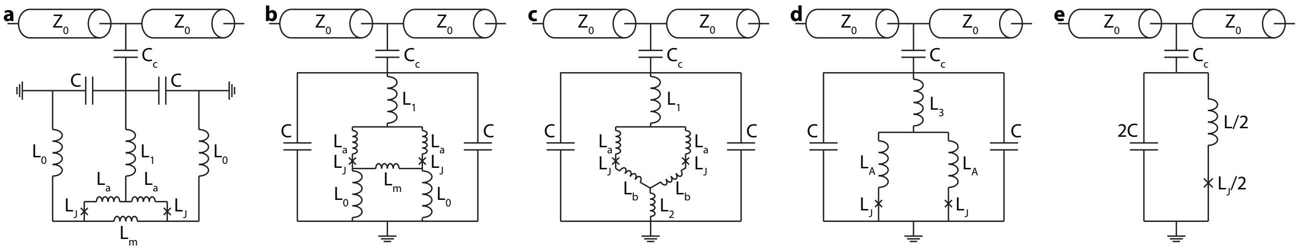

SUPPLEMENTARY FIGURE 3. **Schematic of the device circuit and its simplification.** **a** The device equivalent circuit with individual circuit elements for each geometric element. **b** Re-arrangement of the circuit elements with a single ground connection. **c** Transformation of the inductors L_0, L_m to L_2, L_b using the Δ−Y-approach for impedance-bridges. **d** Combining series inductors into single inductors L_3 and L_A. **e** Combining parallel elements to get the reduced circuit equivalent.

3. Analytical cavity model

The cavity used in this experiment is a lumped element SQUID cavity capacitively coupled to a transmission line through a coupling capacitor C_c. Supplementary Fig. 3 shows a circuit equivalent of the cavity including the coupling capacitor and the feedline with characteristic impedance Z_0. In **a**, a circuit equivalent is shown, which resembles the geometric cavity elements. To get a simplified circuit, we first transform the inductances L_m, L_0 forming an inductance-bridge via the Δ−Y-approach to the new equivalent inductors

$$L_b = \frac{L_0 L_m}{2L_0 + L_m} \tag{3}$$

$$L_2 = \frac{L_0^2}{2L_0 + L_m} \tag{4}$$

and then combine series and parallel elements to arrive with the simple circuit equivalent shown in Supplementary Fig. 3 **e**. The additional relations between the inductors given in **a** and **e** are given by

$$L = L_A + 2L_3 \tag{5}$$

$$L_A = L_a + L_b \tag{6}$$

$$L_3 = L_1 + L_2. \tag{7}$$

As values for our device we estimate $L_0 = 1\,\mathrm{nH}$, $L_1 = 140\,\mathrm{pH}$, $L_m = 60\,\mathrm{pH}$ and $L_a = 45\,\mathrm{pH}$. We estimate the critical currents of our Josephson junctions $I_c = 25\,\mu\mathrm{A}$, which corresponds to a Josephson inductance of $L_J = 13\,\mathrm{pH}$.

Thus, the total inductance of the circuit is given by $L_\mathrm{tot} = (L + L_J)/2$ and the total capacitance by $C_\mathrm{tot} = 2C + C_c$.

4. Characteristic feedline impedance and external linewidth

The external linewidth κ_e of the circuit shown in Supplementary Fig. 3**e** is given by

$$\kappa_e = \frac{\omega_0^2 C_c^2 Z_0}{2C_\mathrm{tot}} \tag{8}$$

which for our device and a feedline impedance of $Z_0 = 50\,\Omega$ predicts $\kappa_e = 2\pi \cdot 3.5\,\mathrm{MHz}$. This is in slight disagreement with the experimentally determined linewidth of about 9 MHz around the flux sweetspot, which can be explained by a combination of two effects. First, the on-chip feedline was designed to have a geometric characteristic impedance $Z_{0g} = 50\,\Omega$, which is considerably increased due to the high kinetic inductance of the thin Aluminum film. And secondly, we have strong cable resonances in the setup on the order of 2 dB peak-to-peak amplitude. Both effects considerably modify the effective impedance attached to the circuit.

When the cavity resonance frequency is tuned and moves through the cable resonances, we also find that the (external) linewidth considerably reduces to about $\kappa = 2\pi \cdot 5\,\mathrm{MHz}$, cf. Supplementary Fig. 7.

5. Intracavity photon number

The photon number in the cavity is estimated using

$$n_c = \frac{2P_{\text{in}}}{\hbar\omega_d}\frac{\kappa_e}{\kappa^2 + 4\Delta^2}, \tag{9}$$

where P_{in} is the input power (in Watt) on the feedline, ω_d is the frequency of the drive tone and $\Delta = \omega_d - \omega_0$ the detuning from the cavity resonance. Note, that we use $\kappa_e = \kappa$ for this estimation as the device is highly overcoupled.

Response function and fitting routine

1. The ideal cavity response function

The S_{21}^{ideal} response function of a parallel LC circuit capacitively side-coupled to a transmission line is given by

$$S_{21}^{\text{ideal}} = 1 - \frac{\kappa_e}{\kappa_i + \kappa_e + 2i\Delta} \tag{10}$$

with internal and external decay rates

$$\kappa_i = \frac{1}{RC_{\text{tot}}}, \qquad \kappa_e = \frac{\omega_0^2 C_c^2 Z_0}{2C_{\text{tot}}} \tag{11}$$

and detuning from the resonance frequency

$$\Delta = \omega - \omega_0, \qquad \omega_0 = \frac{1}{\sqrt{L_{\text{tot}}C_{\text{tot}}}} \tag{12}$$

2. The real cavity response function

The presence of attenuation, cable resonances and parasitic transmission channels is usually captured by additional terms added and multiplied to the ideal cavity response function

$$S_{21} = A\left(S_{21}^{\text{ideal}} + Be^{i\beta}\right)e^{i\alpha} \tag{13}$$

where A, B, α, β are possibly frequency-dependent factors changing the overall transmission function. This can also be written as

$$S_{21} = P\left(1 - \frac{Ke^{i\theta}}{\kappa + 2i\Delta}\right)e^{i\phi} \tag{14}$$

where K and θ are functions of κ_e, B and β and P and ϕ are functions of A, B, α and β. Equation (14) is used throughout this work for fitting the cavity response function and to extract the total linewidth and resonance frequency. Note that a reliable extraction of external and internal linewidths is not possible anymore in the presence of cable resonances and parasitic transmission channels.

3. Full cavity fitting routine

During the experiments, the transmitted signals suffer from interferences and losses due to the presence of microwave elements such as attenuators, circulators and amplifiers in the lines, cf. Supplementary Fig. 2 as well as additional losses from microwave cables. For fitting and calibrating the transmitted fields, we follow a step-by-step fitting routine, which is described as follows. First, we consider the presence of a frequency dependent background signal expressed as

$$S_{\text{back}} = P(\omega)e^{i\phi(\omega)}. \tag{15}$$

For the experimental extraction of the background curve, the cavity is initially set to two distant flux bias points with frequencies $\omega_1 = 2\pi\cdot 5.15$ GHz and $\omega_2 = 2\pi\cdot 5.22$ GHz and afterwards the spectrum is reconstructed by combining

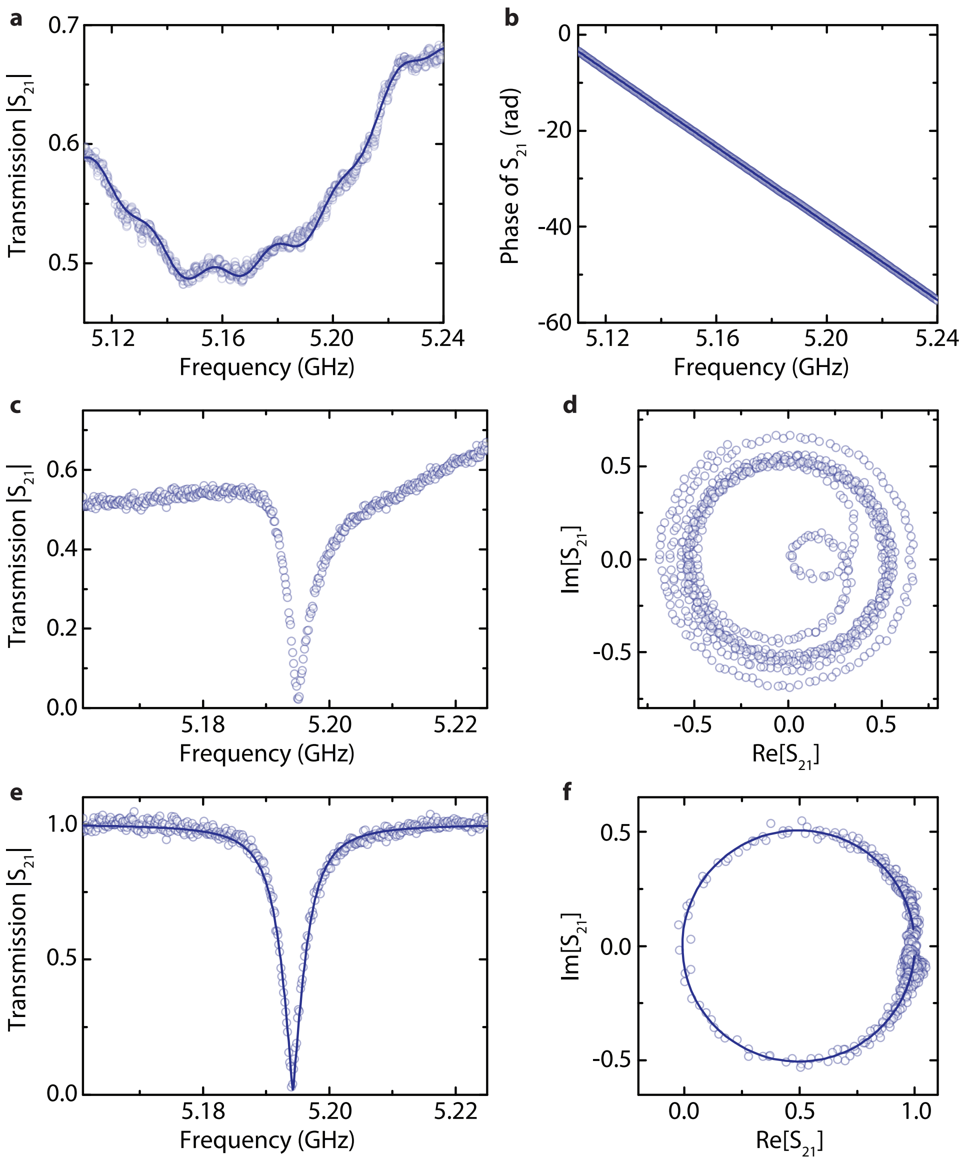

SUPPLEMENTARY FIGURE 4. **The background transmission and how we correct for it.** **a** Background transmission signal amplitude in the relevant frequency range obtained by tuning the cavity to the maximum and minimum frequency and stitching together the unperturbed parts of the background signal. The corresponding phase is shown in **b**. Circles show measurement data, lines are fits as described in the text. **c** and **d** show the response signal of the cavity in raw data. The amplitude is shown in **c** and the complex response in **d**. By means of complex division, we divide off the background fit curves obtained from **a** and **b**. The resulting curve is fitted by Eq. (18). After this fit, we rotate and rescale the cavity resonance and obtain the signal shown in **e** and **f** as circles. The lines show the accordingly rescaled and rotated fits.

the individual parts where the cavity is non-resonant. The amplitude and phase data obtained by this procedure are shown in Supplementary Fig. 4**a** and **b** as circles. Then, we fit the whole background with a complex function whose magnitude and phase are written as a function of frequency as

$$P(\omega) = a_p\omega^5 + b_p\omega^4 + c_p\omega^3 + d_p\omega^2 + e_p\omega + f_p + a_{1c}\cos(b_{1c}\omega + c_{1c}) + a_{2c}\cos(b_{2c}\omega + c_{2c}) \tag{16}$$

$$\phi(\omega) = a_\phi\omega + b_\phi, \tag{17}$$

i.e., we perform a linear fit to the phase and both a polynomial and cosine fit to the magnitude of the stitched background data. The corresponding fits are shown as lines in Supplementary Fig. 4**a** and **b**.

A measured transmission spectrum with the cavity resonance included is shown in Supplementary Fig. 4**c** and **d**. Prior to all cavity fits and fits of optomechanically induced transparency, we remove the reconstructed background signal from the measured signal by complex division.

Considering the possibility that the measured signal might still be influenced by a small frequency-dependent background modulation, we fit the resulting cavity line with

$$S_{21} = (a_{p2} + b_{p2}\omega)\left(1 - \frac{Ke^{i\theta}}{\kappa + 2i\Delta}\right)e^{i(a_{\phi 2}\omega + b_{\phi_2})} \tag{18}$$

where we consider once more a background using the complex scaling factor

$$S_{\mathrm{back2}} = (a_{p2} + b_{p2}\omega)e^{i(a_{\phi 2}\omega + b_{\phi_2})}. \tag{19}$$

Supplementary Figs. 4**e** and **f** show a resonance curve of the SQUID cavity after background division and rotation by the obtained value of θ including the cavity response fit using Eq. (18).

The Josephson junctions and the SQUID

1. The junctions

The constriction type Josephson junctions in our SQUID are designed to be 50 nm wide and 200 nm long nanobridges in between two superconducting pads, similar to what has been investigated previously by other authors[2]. The pads and the junctions have a constant film thickness of about 20 nm and thus we have what is referred to as 2D SQUID geometry in literature[3]. We estimate the critical current to be approximately $I_{c0} \approx 25\,\mu$A. Although our junctions might show deviations from an ideal sinusoidal current-phase relation[3], we can estimate the zero-bias junction inductance from the critical current to be

$$L_J = \frac{\Phi_0}{2\pi I_c} \approx 13\,\mathrm{pH}. \tag{20}$$

2. The SQUID loop inductance

Due to the 2D SQUID geometry as well as the large kinetic inductance of our films, we have to consider a significant loop inductance when treating the SQUID. From our estimations above, the loop inductance is approximately given by $L_l = 2L_a + L_m \approx 150\,\mathrm{pH}$, which gives for the so-called screening parameter

$$\beta_L = \frac{2I_{c0}L_l}{\Phi_0} \approx 3.7. \tag{21}$$

Such a large screening parameter is related to a hysteretic flux state of the SQUID and allows the SQUID to screen more than half a flux quantum before the critical current of the junctions is exceeded by the screening current[4].

3. Bias flux dependence of the resonance frequency

Both, non-sinusoidal current-phase relationship in the form of skewed sine functions as well as large screening parameters lead to widening of the magnetic flux arch and to hysteretic switching of the SQUID flux state. Both descriptions have been used to model the hysteretic resonance frequency flux archs of superconducting resonators including SQUIDS with constriction type Josephson junctions[2,4].

We phenomenologically include both effects in the description of the single-arch flux-dependence of our SQUID cavities by including a factor γ_L into the effective single junction inductance

$$L_J(\Phi) = \frac{L_{J0}}{\cos\left(\pi\gamma_L\frac{\Phi}{\Phi_0}\right)}. \tag{22}$$

The factor γ_{L} takes a widening of the flux arch and a tuning of the resonance frequency far beyond $\pm\Phi_0/2$ into account, cf. Supplementary Fig. 5, where an ideal SQUID with a sinusoidal current-phase relationship and negligible loop inductance would have $\gamma_L = 1$. The resonance frequency of the SQUID cavity can therefore be expressed as

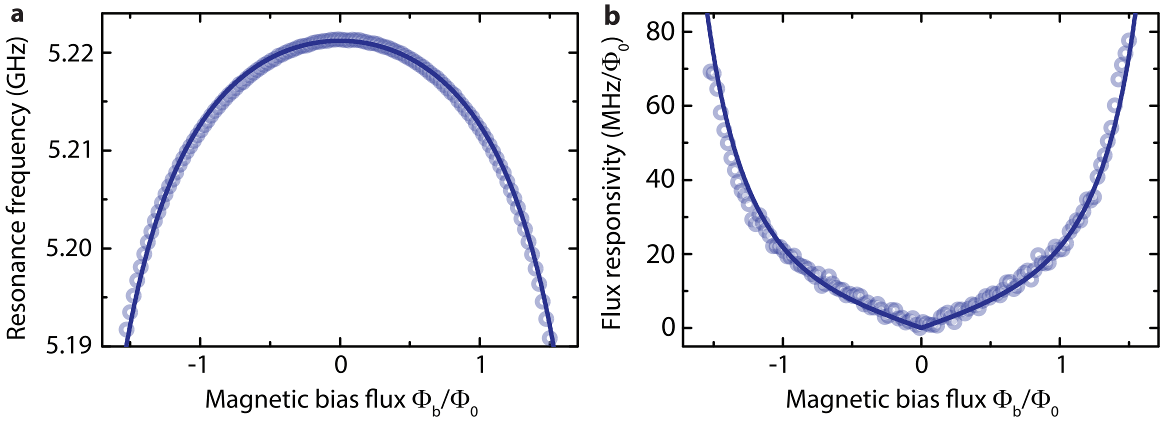

SUPPLEMENTARY FIGURE 5. **Cavity frequency tuning and flux responsivity with magnetic bias flux. a** Cavity resonance frequency vs magnetic bias flux for $B_{||} = 3\,\mathrm{mT}$. Circles are data points extracted from fits and the line is a fit using Eq. (25) with fixed $\Lambda = 0.99$ and γ_L being the only free parameter. In **b** the flux responsivity $|\partial\omega_0/\partial\Phi|$ is plotted. Both, the experimental and the theoretical curves are obtained by calculating the derivative of the data in **a**.

$$\omega_0(\Phi) = \frac{1}{\sqrt{C_\mathrm{tot}(L + L_J(\Phi))/2}}. \tag{23}$$

Defining the sweet spot resonance frequency by

$$\omega_0^s = \frac{1}{\sqrt{C_\mathrm{tot}(L + L_{J0})/2}} \tag{24}$$

we can write the flux-dependent frequency as

$$\omega_0(\Phi) = \frac{\omega_0^s}{\sqrt{\Lambda + \frac{1-\Lambda}{\cos\left(\pi\gamma_L\frac{\Phi}{\Phi_0}\right)}}}. \tag{25}$$

with $\Lambda = L/(L + L_{J0})$. For our device parameters, we get $\Lambda \approx 0.99$.

Supplementary Fig. 5**a** shows the resonance frequency of the SQUID cavity when biased with the on-chip bias line and the resulting flux arch was fitted with Eq. (25). The only free parameter for the fit was $\gamma_L = 0.23$, indicating a large screening parameter and/or a non-sinusoidal current-phase relation. We note here, however, that the theoretical $\beta_L = 3.7$ derived above is too small to explain the widening of the arch as we observe it. One possible explanation is a non-sinusoidal current-phase relation. A second possibility, which is at the same time in agreement with the deviation between theory and experiment of the mechanical resonance frequency shift with in-plane field, is that we underestimate the loop inductance significantly. A discussion of this possibility with a possible explanation is given in Supplementary Note 4.

In Supplementary Fig. 5**b**, we plot the derivative of both, the data points and the fit curve, to obtain the flux responsivity $\partial\omega_0/\partial\Phi$, which is directly proportional to the optomechanical single-photon coupling rate g_0.

Both parameters, Λ and γ_L seem to depend slightly on the magnetic in-plane field, which is taken into account in our analysis. The values given here are extracted for $B_{||} = 1\,\mathrm{mT}$. The origin of this dependence, however, is not fully clear. It might be due to a change of the bias current flow for large in-plane fields or to a change of kinetic loop inductance, while the rest of the kinetic cavity inductance stays nearly unchanged.

4. Calibration of the flux axis

We use the measured Φ_0-periodicity of jumps in the hysteretic resonance frequency to calibrate the flux axis for the bias flux dependence.

Supplementary Fig. 6 shows an example for the hysteretic jumps of the cavity frequency with flux, indicating a significant loop inductance and/or a non-sinusoidal current-phase relation[2,4]. We herewith calibrate the flux axis for

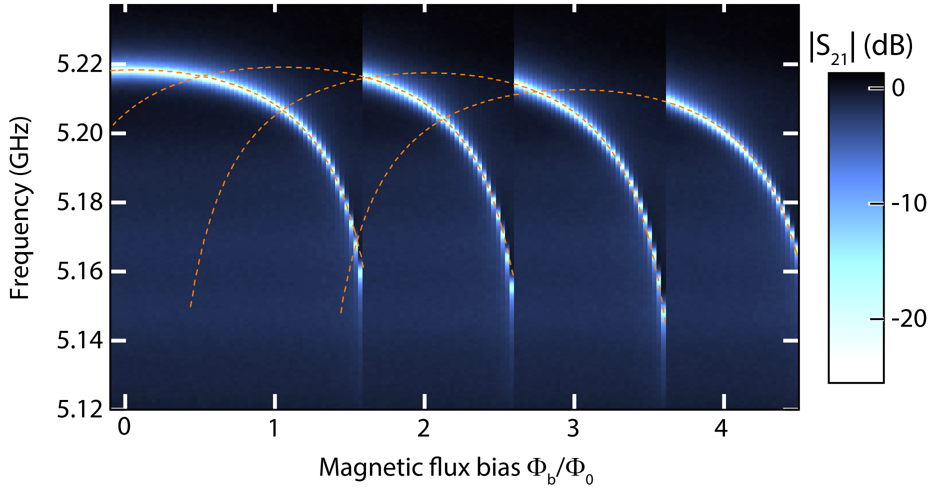

SUPPLEMENTARY FIGURE 6. **Cavity frequency tuning with magnetic bias flux beyond a single flux arch.** When we sweep the bias flux to larger values than about $1.6\Phi_0$, we find periodic jumps in the resonance frequency and partial archs. This is an indication for a non-negligble screening parameter and/or a non-sinusoidal current-phase relation. The periodicity can be used to calibrate the flux axis to the flux quantum Φ_0. The dashed lines correspond to copies the flux arch dependence used in Supplementary Fig. 5**a** each shifted in flux and sweetspot frequency only to match the observed resonance frequencies. The data shown here are for $B_{||} = 1\,$mT

all in-plane fields. Note, that in contrast to the description given in Ref.[4], the periodicity of the jumps corresponds to $1\Phi_0$ instead of $2\Phi_0$.

Also, we note here that according to this flux calibration based on the periodicity of the resonance frequency, the experimentally obtained current-to-flux conversion $40\,\mu\text{A}\widehat{=}1\Phi_0$ is not in agreement with what we would expect from calculating the flux in the SQUID loop generated by a flux bias line current $I_b = 40\,\mu$A. We suspect that the bias current is flowing to ground through the SQUID and the mechanical oscillator itself, which is possible as all corresponding wires, the linear inductors of the cavity as well as the bias current, are galvanically connected through the superconducting ground planes to each other.

5. In-plane field dependence of the cavity parameters

The cavity parameters of the SQUID cavity such as the linewidth κ and the shape of the flux dependence depend on the magnetic in-plane field. In Supplementary Fig. 7 we show exemplary results for the cavity resonance frequency in **a** and the cavity linewidth in **b** vs the bias flux and for several magnetic in-plane fields. Due to the fact, that the parameters depend only slightly on the magnetic in-plane field for $B_{||} \leq 7\,$mT, we only plot a subset of curves from this regime here and add the two curves for $B_{||} = 9\,$mT and $10\,$mT, where we observe systematic deviations.

Besides different frequencies at the flux sweet spot, the bias flux dependences for $B_{||} = 1, 4, 7\,$mT are very similar. For 9 and $10\,$mT, however, a slight widening of the arch is visible. For now, it is unclear to us what exactly causes this widening. Some effects such as a global increase of kinetic inductance or a global Abrikosov vortex pollution can probably be disregarded, as we expect that they would significantly impact the total cavity resonance frequency and/or linewidth.

In **b** we plot the extracted total cavity linewidth, which is dominated by the external linewidth for all shown values. We observe a strong linewidth decrease by about a factor of 2 with bias flux and only small variations due to the in-plane field. We attribute this linewidth decrease with bias flux not to the bias flux directly, but think it is related to the frequency position of the cavity within a standing wave pattern of cable and feedline resonances in the setup. In panel **c** we plot the same linewidth data for all in-plane fields vs the cavity resonance frequency and find that besides a very slight dependence on in-plane field all points fall onto the same curve. We plot in panel **c** as black curve the background transmission signal discussed in Supplementary Fig. 4, indicating that linewidth and background transmission are correlated. We notice in particular that the linewidth increases again for the smallest frequency points around the value where the background transmission increases again as well.

Simulations with the package QUCS confirm the possibility of a significant linewidth dependence on the resonance frequency in presence of feedline resonances. An intuitive picture would be that by shifting the cavity resonance frequency with bias flux, it is moved from an antinode to a node of a standing wave formed on the feedline.

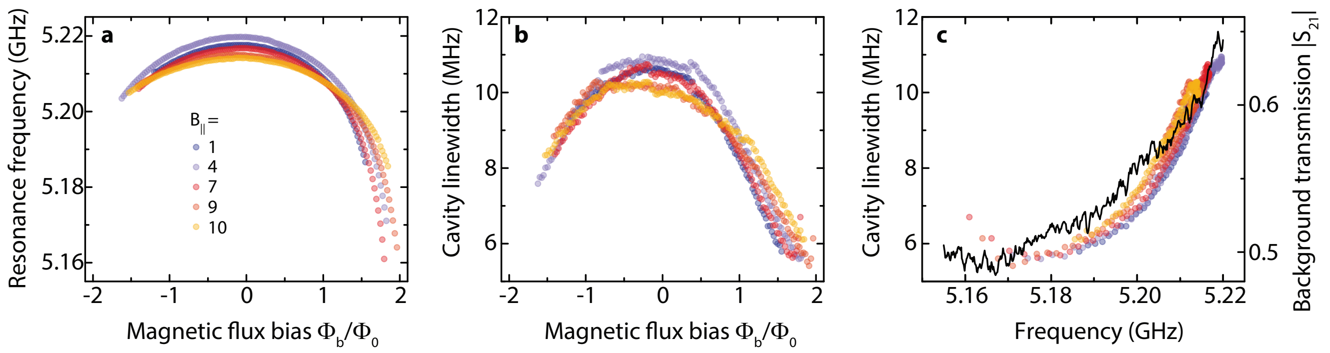

SUPPLEMENTARY FIGURE 7. **Bias flux dependence of the cavity parameters and their development with in-plane field. a** SQUID cavity resonance frequency vs magnetic bias flux for 5 different magnetic in-plane fields. **b** Cavity linewidth vs magnetic bias flux for 5 different magnetic in-plane fields. **c** Cavity linewidth plotted vs the simultaneously fitted resonance frequency for 5 different magnetic in-plane fields. The black line shows the background transmission amplitude in the corresponding frequency range, showing a very similar trend as the cavity linewidth. The color legend for the data points in all three plots is given in **a**. Data in **a** and **b** have been shifted in flux such that the sweetspot is positioned at $\Phi_b = 0$.

As concluding remarks for this part we would like to state, that we compensate for a flux arch widening at the highest fields by adjusting the flux bias point to the desired $\partial\omega_0/\partial\Phi$. Also, the sweetspot resonance frequency and linewidth tend to be influenced by the magnetic history and by microwave triggered flux avalanches. They can vary by several MHz for a given in-plane field, indicating that we trap flux in form of Abrikosov vortices inside the sample originating from the out-of-plane component of the transduction field. Thermal cycling before each measurement could partially overcome this issue, but is too time consuming to be a practical solution.

6. Cavity anharmonicity

Assuming a sinusoidal current-phase relation, we calculate the shift per photon to first order by

$$\chi = -\frac{e^2}{2\hbar C_{\mathrm{tot}}}\left(1-\Lambda\right)^3 \approx 2\pi \cdot 14\,\mathrm{Hz}. \tag{26}$$

Therefore, the cavity can be considered in good approximation as linear, as long as the photon number does not exceed a few 10000.

SUPPLEMENTARY NOTE 4: MECHANICAL CHARACTERIZATION

Theory of Lorentz-force actuation

The equation of motion of the mechanical resonator is given by

$$\ddot{x} + \frac{\Omega_0}{Q_m}\dot{x} + \Omega_0^2 x = \frac{F(t)}{m} \tag{27}$$

where m is the effective mass, Q_m is the mechanical quality factor and Ω_0 is the resonance frequency. External forces onto the mechanical oscillator are contained in $F(t)$.

The current through the mechanical beam in presence of flux biasing and a magnetic in-plane field is given by the flux quantization and conservation in the SQUID loop. In the absence of a bias current and for identical junctions, the general relation between the phase difference across one junction δ and the total flux through the loop Φ is given by

$$\frac{\delta}{\pi} = \frac{\Phi}{\Phi_0}. \tag{28}$$

The circulating current at the same time is related to the phase difference by

$$J = I_{c0}\sin\delta \tag{29}$$

The total flux through the loop Φ is a sum of the bias flux Φ_b, the flux generated by a loop current via the loop inductance $\Phi_J = L_l J$, and a contribution from the in-plane field when the mechanical oscillator is displaced from its equilibrium position $\Phi_x = \gamma B_{||} l x$, thus

$$\Phi = \Phi_b + \Phi_J + \Phi_x \tag{30}$$
$$= \Phi_b + L_l J + \gamma B_{||} l x. \tag{31}$$

For a constant flux bias Φ_{b0} there is a circulating current J_0 and the mechanical beam is in the equilibrium position x_0. We assume now that all quantities only slightly differ from their equilibrium values $\Phi_b(t) = \Phi_{b0} - \Delta\Phi_b$, $x(t) = x_0 + \Delta x$ and $J(t) = J_0 + \Delta J$. Redefining $x = \Delta x$ and $L_J = L_J(\Phi_{b0})$, we can approximate to first order

$$\Delta J = \frac{\Delta\Phi_b}{L_l + 2L_J} - \frac{\gamma B_{||} l x}{L_l + 2L_J}. \tag{32}$$

The dynamical part of the Lorentz-force is given by $F_\mathrm{L}(t) = \gamma B_{||} l \Delta J$ and thus the equation of motion becomes

$$\ddot{x} + \frac{\Omega_0}{Q_m}\dot{x} + \left(\Omega_0^2 + \frac{\gamma^2 B_{||}^2 l^2}{m(L_l + 2L_J)}\right) x = \frac{\gamma B_{||} l}{m(L_l + 2L_J)} \Delta\Phi_b(t). \tag{33}$$

Thus, a time-varying magnetic flux is translated into a time-varying Lorentz-force and can be used to directly drive the mechanical motion. In addition, a position-dependent force emerges from the mechanical oscillator placed in a SQUID loop, which shifts the mechanical resonance frequency.

In-plane magnetic field dependence

The position dependent part of the Lorentz-force is equivalent to a mechanical spring stiffening, in analogy to the electrostatic softening in electromechanical capacitors. The shifted resonance frequency is given by

$$\Omega_m^2 = \Omega_0^2 + \frac{\gamma^2 B_{||}^2 l^2}{m(L_l + 2L_J)} \tag{34}$$

what can be approximated as

$$\Omega_m \approx \Omega_0 + \frac{\gamma^2 B_{||}^2 l^2}{2m\Omega_0(L_l + 2L_J)}. \tag{35}$$

We indeed observe a shift of the mechanical resonance frequency with in-plane field as shown in Supplementary Fig. 8 for two different flux responsivities, i.e., for two different Josephson inductances. The absolute numbers, however, are smaller by about a factor of ~ 2 than the result of independent calculations based on the device parameters and the in-plane field. Possible reasons for this mismatch is the overestimation of the mode scaling factor $\gamma = 0.86$, which we determined through matching the experimental g_0 with the theoretical calculations, an underestimated loop inductance or a field-dependent loop or Josephson inductance.

In combination with the observation of flux arch widening, we consider the most probable explanation that the loop inductance is significantly higher than expected. For the mechanical resonance frequency shift, we find a good agreement between theory and experiment for a loop inductance of $\sim 350\,\mathrm{pH}$. This would correspond to $\beta_L = 8.6$. A possible origin for this deviation is possibly related to the suspension of the mechanical part of the loop and the release process, which ends with oxygen plasma ashing of the resist and might induce an enhanced oxidation of the bottom side of the beam. The mechanical beam oxidizes from the top and the bottom, while the rest of the circuit only oxidizes from the top. For very thin films as used here, the oxide layer of a few nm thickness might change the thickness of the superconducting layer significantly, which will increase the kinetic inductance of that region. As the inductance of our circuit is dominated by kinetic inductance anyways, such a two-sided oxidization might indeed be responsible for a significantly increased inductance of the suspended parts. This would explain, why the results related to the loop inductance are deviating from theoretical calculations, while all results where the loop inductance is not relevant, are in excellent agreement.

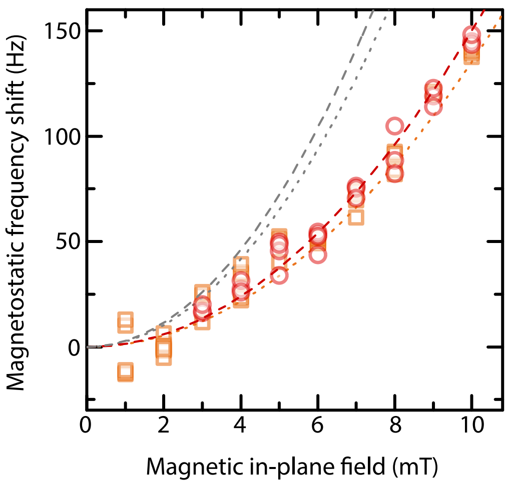

SUPPLEMENTARY FIGURE 8. **Magnetostatic spring stiffening by Lorentz-force backaction.** The measured frequency shift is plotted as points for two different values of bias flux. Circles corrrespond to $\Phi_b/\Phi_0 = 0.75$ and squares to $\Phi_b/\Phi_0 = 1.45$. The gray dashed and dotted lines are the theoretical calculations without free parameters and overestimate the measured effect by a factor of ~ 2. The red dashed and orange dotted lines correspond to the theoretical lines with a scaling factor of ~ 0.52 and agree well with the observed frequency shift.

Upconversion of coherently driven mechanical motion

We excite the mechanical resonator by Lorentz-force actuation and measure the cavity sidebands generated by the corresponding cavity field phase modulation when sending a tone resonant with the cavity $\omega_d = \omega_0$. The excitation current is generated by the output port of a vector network analyzer and sent through the on-chip bias line, cf. Supplementary Fig. 2**c**. At the same time, we drive the cavity with a resonant microwave tone generated by a signal generator. The cavity output field, including the motional sidebands, is amplified and sent through a high-pass filter into a mixer, where it is down-converted by being mixed with the original carrier tone. The mixer output is low-pass filtered and sent into the input port of the network analyzer. As we are driving the cavity on resonance, we must adjust the phase of the carrier signal in order to get constructive interference of the sidebands at $+\Omega$ and $-\Omega$. We adjust the phase-shifter manually until the detected sideband signal is maximized.

In this setup, however, we do not only detect the additional flux induced into the SQUID by the mechanical motion, but also the phase modulations directly generated by the bias flux modulation itself. Other possible parasitic tones come from mixing due to the cavity nonlinearity or in the nonlinear elements of the detection chain. The detected sideband amplitude $|S_{21}|$ is thus proportional to

$$|S_{21}(\Omega)| \propto \left| \frac{\gamma B_{||} l}{2m\Omega_m} \frac{F_{\mathrm{L}}(\Omega)}{\Omega_m - \Omega - i\frac{\Gamma_m}{2}} + Se^{i\sigma} \right| \tag{36}$$

with an additional signal $Se^{i\sigma}$ interfering with the motional sideband. Therefore, the measured, upconverted mechanical resonance will have a slight Fano lineshape as shown in Supplementary Fig. 9**a**. We correct for this slight asymmetry by substracting a constant complex number from the detected signal. The result is shown in Supplementary Fig. 9**b** and in Fig. 2 of the main paper.

Interferometric detection of thermal mechanical motion

The measurement routine is very similar to the one for the detection of coherently driven motion. Instead of using a network analyzer, however, we do not apply any driving current, but just detect the down-converted sideband-voltage quadratures I and Q with a vector signal analyzer. From the Fourier-transform of the quadratures, we calculate the corresponding power spectral density.

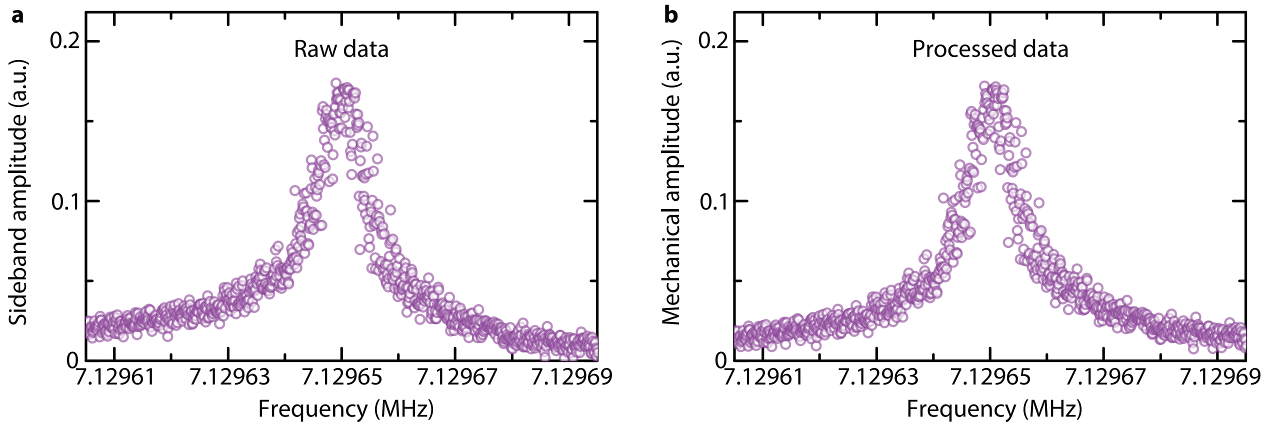

SUPPLEMENTARY FIGURE 9. **Processing the motional sideband generated by mechanical displacement.** **a** Raw data for the sideband amplitude detected by means of sending a resonant tone into the cavity while exciting the mechanical mode by Lorentz-force. The Lorentz-force drive current frequency is swept through the mechanical resonance. Due to additional contributions to the SQUID cavity sideband such as direct flux modulation of the SQUID by the Lorentz-force current, the sideband does not only contain information about the mechanical displacement. **b** shows the amplitude data of **a**, where a constant complex number has been substracted from the complex S_{21} data.

Estimate of the thermal phonon occupation

We assume the SQUID cavity to have negligible thermal occupation at the fridge base temperature $T_b = 15\,\mathrm{mK}$ and the thermal occupation of the mechanical mode to be $n_{\mathrm{th}} \gg 1$. Then, for a resonant cavity drive, the power spectral density of the system output is approximately given by

$$\frac{S(\omega)}{\hbar\omega} = \frac{1}{2} + n'_{\mathrm{add}} + 2\mathcal{C}\kappa_e \frac{\kappa}{\kappa^2 + 4\Omega_m^2} \frac{\Gamma_m^2}{\Gamma_m^2 + 4\Delta_m^2} n_{\mathrm{th}} \tag{37}$$

where n'_{add} is the effective number of added noise photons and in our case is mainly determined by the HEMT noise temperature $T_{\mathrm{HEMT}} \approx 2\,\mathrm{K}$ and the attenuation of $2\,\mathrm{dB}$ between the sample output and the HEMT amplifier. From these numbers, we estimate $n'_{\mathrm{add}} \sim 14$.

With the cooperativity $\mathcal{C} \approx 0.2$ and $\kappa_e \approx \kappa \approx \Omega_m$, we calculate from this $n_{\mathrm{th}} \approx 160$ for the measurement shown in the main paper. This occupation corresponds to a mode temperature of $n_{\mathrm{th}} \approx 50\,\mathrm{mK}$ and suggests that the mechanical oscillator is not fully thermalized to the fridge base temperature. We also note, however, that the estimation of this number has several significant uncertainties related to the phase of the local oscillator in our homodyne detection scheme (manually adjusted), to the possibility that the cavity field emission is not symmetric into both sides of the transmission line and to small uncertainties related to the parameters $\mathcal{C}$ and κ.

SUPPLEMENTARY NOTE 5: OPTOMECHANICAL DEVICE CHARACTERIZATION

Optomechanical equations of motion

The system is modelled with the classical equations of motion for the mechanical displacement x and normalized intracavity field amplitude α

$$\ddot{x} = -\Gamma_m \dot{x} - \Omega_m^2 x + \frac{1}{m}(F_r + F_e) \tag{38}$$

$$\dot{\alpha} = \left[i(\Delta + Gx) - \frac{\kappa}{2} \right] \alpha + \sqrt{\frac{\kappa_e}{2}} S_{\mathrm{in}}, \tag{39}$$

where $\Delta = \omega_d - \omega_0$ is the detuning from the cavity resonance frequency, $\kappa = \kappa_i + \kappa_e$ is the total cavity linewidth and S_{in} is the normalized input field. The external forces onto the mechanical oscillator are expressed by F_e and the

radiation pressure force contribution is taken into account in F_r and expressed as a function of the intracavity field by

$$F_r = \hbar G \left|\alpha\right|^2, \tag{40}$$

with pull parameter G

$$G = -\frac{\partial \omega_0}{\partial x}. \tag{41}$$

Assuming that the intracavity field is high enough to only consider small deviations from the steady state solutions with $x = \bar{x} + \delta x$ and $\alpha = \bar{\alpha} + \delta\alpha$ and no external driving force F_e, the equations of motion can be linearized as

$$\delta\ddot{x} = -\Gamma_m \delta\dot{x} - \Omega_m^2 \delta x + \frac{\hbar G \bar{\alpha}}{m}(\delta\alpha + \delta\alpha^*) \tag{42}$$

$$\delta\dot{\alpha} = \left[i\bar{\Delta} - \frac{\kappa}{2}\right]\delta\alpha + iG\bar{\alpha}\delta x + \sqrt{\frac{\kappa_e}{2}} S_p \tag{43}$$

In the above expressions, the detuning $\bar{\Delta} = \omega_d - \omega_c + G\bar{x}$ takes into account the shift from the equilibrium position $\bar{x}$ due to the radiation pressure force and $\sqrt{\frac{\kappa_e}{2}} S_p$ with $S_p = S_0 e^{-i\Omega t}$, $\Omega = \omega - \omega_d$ accounts for field fluctuations. As in our experiments $\bar{\Delta} \approx \Delta$, we will just use Δ instead of $\bar{\Delta}$ throughout this paper.

The response of the optomechanical cavity is then given by

$$S_{21} = 1 - \sqrt{\frac{\kappa_e}{2}} \frac{a_-}{S_0} \tag{44}$$

with

$$a_- = \chi_c \left[1 + i2m\Omega_m g^2 \chi_c \chi_m^{\text{eff}}\right] \sqrt{\frac{\kappa_e}{2}} S_0. \tag{45}$$

Here

$$\chi_c = \frac{1}{\frac{\kappa}{2} - i(\Delta + \Omega)} \tag{46}$$

is the cavity susceptibility and

$$\chi_m^{\text{eff}} = \frac{1}{2m\Omega_m} \frac{1}{\Omega_m - \Omega - i\frac{\Gamma_m}{2} + \Sigma(\Omega_m)} \tag{47}$$

with

$$\Sigma(\Omega_m) = -ig^2 \left[\chi_c(\Omega_m) - \chi_c^*(-\Omega_m)\right] \tag{48}$$

is the effective mechanical susceptibility in the high-Q_m approximation.

Optical spring and optical damping

By re-writing Eq. (48) as $\Sigma = \delta\Omega_m - i\Gamma_0/2$ and analyzing the real and imaginary part we can write the change in mechanical frequency $\delta\Omega_m$ (optical spring) and the additional mechanical damping term Γ_o (optical damping) as

$$\delta\Omega_m = g^2 \left[\frac{\Delta + \Omega_m}{\frac{\kappa^2}{4} + (\Delta + \Omega_m)^2} + \frac{\Delta - \Omega_m}{\frac{\kappa^2}{4} + (\Delta - \Omega_m)^2}\right] \tag{49}$$

$$\Gamma_o = g^2 \kappa \left[\frac{1}{\frac{\kappa^2}{4} + (\Delta + \Omega_m)^2} - \frac{1}{\frac{\kappa^2}{4} + (\Delta - \Omega_m)^2}\right] \tag{50}$$

For all our experimental parameters, the optical frequency shift is negligibly small $\delta\Omega_m < 1\,\text{Hz}$, i.e., $\delta\Omega_m \ll \Gamma_m$, and therefore is not accounted for in any of the measurements or analyses.

Optomechanically induced transparency in the unresolved sideband regime

For our device, we have $\kappa \sim \Omega_m$ and thus we cannot use the approximate equations and results for the resolved sideband regime. We used two related methods to analyze our experiments on optomechanically induced transparency and to determine the single-photon coupling rate g_0 from these measurements. For a drive on the red sideband and $\Gamma_m \ll \kappa$, both resonances, the cavity response as well as the response window of the mechanical oscillator inside the cavity describe a circle in the complex response. The ratio of the diameters of these circles can be used to determine the optomechanical multi-photon coupling rate g as described below. In the second way, we fit both resonances with a complex resonance function as Eq. (18) and determine the cooperativity from the ratio of the amplitudes on resonance.

1. Cavity circle diameter d_c

To demonstrate that the circle diameter ratio is not influenced by the presence of parasitic resonances and transmission channels of the setup, we start with the modified optomechanical response function similar to what we described above for the bare cavity

$$S_{21} = A\left(1 - \frac{\kappa_e}{\kappa + 2i(\Delta+\Omega)}\left[1 + i2m\Omega_m g^2\chi_c\chi_m^{\text{eff}}\right] + Be^{i\beta}\right)e^{i\alpha} \tag{51}$$

which can be rewritten as

$$S_{21} = P\left(1 - \frac{Ke^{i\theta}}{\kappa + 2i(\Delta+\Omega)}\left[1 + i2m\Omega_m g^2\chi_c\chi_m^{\text{eff}}\right]\right)e^{i\phi}. \tag{52}$$

From the bare cavity fit, we determined the background $S_{\text{back}} = Pe^{i\phi}$ and we divide this background off to get

$$S_{21} = 1 - \frac{Ke^{i\theta}}{\kappa + 2i(\Delta+\Omega)}\left[1 + i2m\Omega_m g^2\chi_c\chi_m^{\text{eff}}\right] \tag{53}$$

For $|\Omega_m - \Omega| \gg \Gamma_m$, the mechanical susceptibility essentially vanishes in the weak coupling limit and we get back the bare cavity response function

$$S_{21} = 1 - \frac{Ke^{i\theta}}{\kappa + 2i(\Delta+\Omega)}. \tag{54}$$

By calculating the cavity response at the points $\Omega = -\Delta - \kappa/2$ and $\Omega = -\Delta + \kappa/2$ we get

$$S_{21-} = 1 - \frac{Ke^{i\theta}}{\kappa + i\kappa}, \qquad S_{21+} = 1 - \frac{Ke^{i\theta}}{\kappa - i\kappa} \tag{55}$$

The distance between these two points gives us the bare cavity circle diameter

$$d_c = |S_{21-} - S_{21+}| = \frac{K}{\kappa}. \tag{56}$$

2. OMIT circle diameter d_m

For the estimation of the diameter of the circle related to the mechanical signal as optomechanically induced transparency (OMIT), we first consider that the anchor point of the mechanical circle does not necessarily correspond exactly to the cavity resonance frequency in order to account for cases where there is still a small detuning present in the experiment. This offset $\delta_m = \omega_0 - \omega_d - \Omega_m$ will modify the diameter of the circle with respect to the resonant case. Considering $\Gamma_{\text{eff}} = \Gamma_m + \Gamma_o \ll \kappa$ we can expect that, for a fixed pump frequency close to the the cavity red sideband $\Delta \approx -\Omega_m - \delta_m$, the cavity has a constant reponse during the OMIT circle, given by

$$\chi_c = \frac{2}{\kappa - 2i\delta_m} \tag{57}$$

By evaluating the total response function at the points $\Omega = \Omega_m - \Gamma_{\text{eff}}/2$ and $\Omega = \Omega_m + \Gamma_{\text{eff}}/2$ we calculate the OMIT circle diameter

$$d_m = |S_{21-} - S_{21+}| = \left| -4iKm\Omega_m g^2 \frac{1}{(\kappa - 2i\delta)^2} \left[\chi_m^{\mathrm{eff}-} - \chi_m^{\mathrm{eff}+} \right] e^{i\theta} \right| \tag{58}$$

$$= 4K \frac{g^2}{\Gamma_{\mathrm{eff}}} \frac{1}{\kappa^2 + 4\delta_m^2}. \tag{59}$$

3. Effective cooperativity $\mathcal{C}_{\mathrm{eff}}$ and the extraction of g_0

We define the effective cooperativity as

$$\mathcal{C}_{\mathrm{eff}} = \frac{4g^2}{\kappa \Gamma_{\mathrm{eff}}} \tag{60}$$

and with this the ratio of the cavity and mechanical resonance circle diameters is given by

$$\frac{d_m}{d_c} = \mathcal{C}_{\mathrm{eff}} \frac{\kappa^2}{\kappa^2 + 4\delta_m^2} \tag{61}$$

Thus, as a measurement of the cavity and the transparency window of OMIT provide us with the circle diameters, the cavity linewidth κ and the detuning δ_m, we can extract the effective cooperativity, which in combination with the width of the transparency window Γ_{eff} allows for the extraction of the multi-photon coupling rate g. Using the estimated intracavity photon number n_c finally leads to the single-photon coupling rate

$$g_0 = \frac{g}{\sqrt{n_c}}. \tag{62}$$

Full experimental and fitting procedure for optomechanically induced transparency

1. Adjusting the cavity parameters and measurement routine

I. As first step in all measurements, we fix the in-plane field to a desired value $B_{||}$.

II. As second step, we sweep the bias flux in small steps and for each value take a transmission spectrum of the cavity with a network analyzer. The cavity resonance is fitted within the measurement script using Eq. (18) and quality factor and resonance frequency are extracted. To approximately bias the cavity with a desired value for $\partial\omega_0/\partial\Phi$, we run this biasing and fitting procedure until the resonance frequency shift between two subsequent bias points matches the set value.

III. Then, we switch on the drive tone at a frequency ω_d slightly below the red sideband frequency $\omega_0 - \Omega_m$ with ω_0 being the last resonance frequency measured in the bias flux sweep, and move the drive tone frequency in small steps towards the cavity resonance frequency. For each pump frequency, we take a resonance curve and extract ω_0 by a fit again, until $\omega_0 - \omega_d - \Omega_m < \kappa/100$, i.e., until the drive tone is approximately on the red sideband.

IV. When this criterion is fulfilled, the iteration stops, we switch off the pulse-tube cooler of the dilution refrigerator and measure one full cavity transmission spectrum as well as a narrow-band zoom-in transmission to the frequency range where the transparency occurs $\Omega \approx \Omega_m$. If we do not switch off the pulse tube cooler, we observe strong frequency fluctuations of the SQUID cavity and often cavity switching out of the metastable into the stable flux branch, which both can be significantly suppressed when the pulse tube cooler is switched off.

This relatively complicated iterative procedure is needed for several reasons. First, due to the non-negligible loop inductance and the possibly non-sinusoidal current-phase relation, we operate the cavity for most measurements in a metastable and hysteretic biasing regime. Second, the cavity resonance frequency depends slightly on the intracavity photon number despite the small anharmonicity. Many parameters such as the flux sweetspot biasing value or the sweetspot frequency depend furthermore slightly on the in-plane field value, what we attribute mainly to an imperfect alignment between sample and magnetic in-plane field, leading to a non-negligible out-of-plane component. Taking all these factors together, a simple fixed biasing procedure to achieve similar parameters for each measurement would not be sufficient.

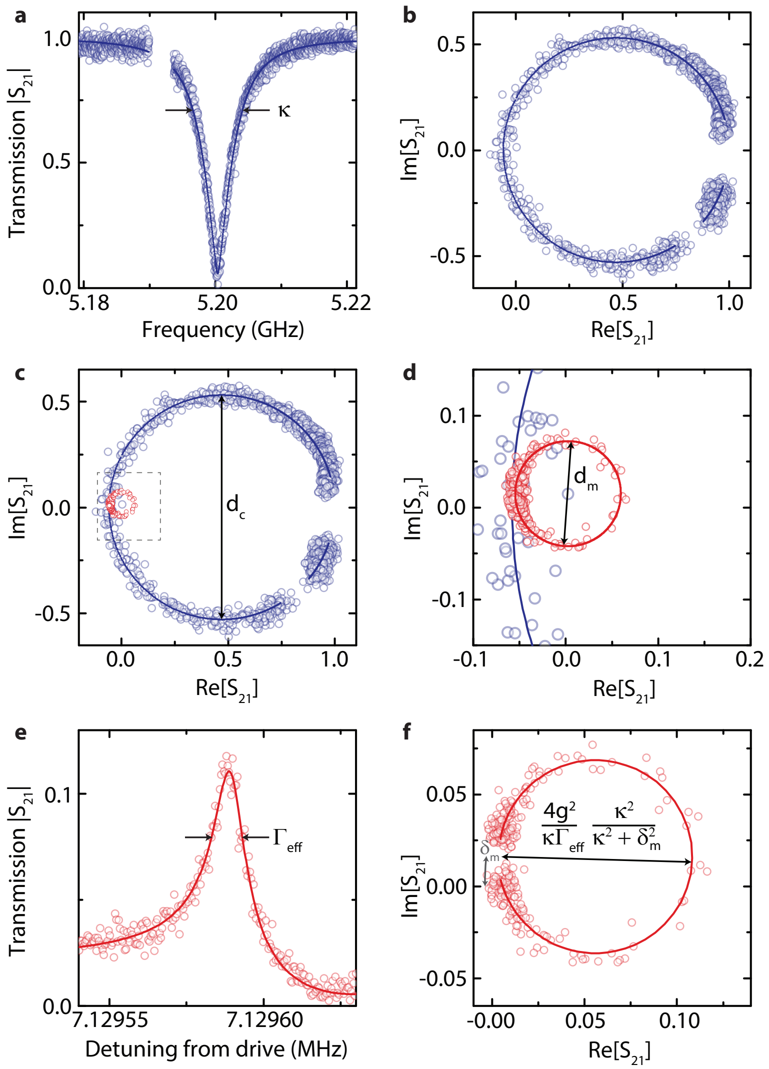

SUPPLEMENTARY FIGURE 10. **Fitting the optomechanical response and extracting the multi-photon coupling rate** $g = \sqrt{n_c} g_0$. **a** Fit of the cavity response amplitude in presence of a red-sideband drive tone. The frequency window of the drive tone is removed for a reliable fitting procedure. **b** Data and fit as in **a**, shown in the complex plane. **c** The large circle corresponds to the cavity response, the small circle to the signal of the optomechanically induced transparency, which is measured separately due to the narrow mechanical linewidth. The diameter of the cavity circle is d_c. The dashed box shows the zoom window plotted in **d**, where the diameter of the OMIT circle is denoted as d_m. In addition to a simple circle fit as represented by the line in **d**, we perform a fit of the complex resonance function to extract the effective mechanical linewidth. The result for the amplitude is shown in **e** and in the complex plane in **f**. Note that the data in **e** and **f** have been shifted and rescaled in the complex plane with respect to **c** and **d**. The scaling has been performed to anker the cavity circle at $S_{21} = 1$ with $d_c = 1$. With this scaling the amplitude of the OMIT response is given by $\frac{4g^2}{\kappa\Gamma_{\mathrm{eff}}}\frac{\kappa^2}{\kappa^2+4\delta_m^2}$ as indicated in **f**. The experimental parameters for this dataset was $B_{||} = 6\,\mathrm{mT}$, $\partial\omega_0/\partial\Phi \sim 2\pi \cdot 17\,\mathrm{MHz}/\Phi_0$ and $n_c \sim 2700$ intracavity photons.

2. Fitting routine

I. For the extraction of the single-photon coupling rate g_0 we initially perform a wide range scan as described in Supplementary Note 3 and get the background fit function $S_{\mathrm{back}} = P(\omega)e^{i\phi(\omega)}$. For all other measurements, we then calculate the complex background signal for the corresponding frequency range and divide it off the data.

II. To fit the resonance curve for each measurement, the pump tone signal, which lies within the cavity line due to $\kappa \sim \Omega_m$, is cut away and the result is fitted as described in Supplementary Note 3 in order to obtain resonance frequency ω_0 and linewdith κ. One example is shown in Supplementary Fig. 10**a** and **b**.

III. For the analysis of the transparency window, once again the backgrounds are divided off in a similar way

as previously done for the cavity. During the cavity fit, the parameters K and θ are determined and the cavity resonance was corrected for them, anchoring the resonance circle at $S_{21} = 1$. In addition, we apply all corrections to the mechanical response as well. An example for the real and imaginary part of both modified cavity and OMIT response functions are shown in Supplementary Fig. 10**c** and **d**. Performing a circle fit as shown in Supplementary Fig. 10**d**, we get the circle diameter d_m.

IV. From a response fit to the mechanical resonance, we finally extract the last missing parameters Γ_{eff} and Ω_m. At this stage, we can also determine the detuning between the cavity resonance frequency and the OMIT resonance δ_m, which can be seen in Supplementary Fig. 10 as slight rotation of the OMIT response along the cavity circle and a Fano-like resonance in Supplementary Fig. 10**e**. For the resonance shown in the main paper Fig. 3, we manually corrected for this rotation.

V. Now we calculate the effective cooperativity and the multi-photon coupling rate g. The single-photon coupling rate g_0 is determined in the last step from g using the independently calculated intracavity photon number n_c.

SUPPLEMENTARY NOTE 6: DATA SCALING, ADDITIONAL DATA AND ERROR BARS

Accounting for deviations in $\partial\omega_0/\partial\Phi$

As mentioned above, we face the complications that the used SQUID cavity is metastable and hysteretic, that the out-of-plane component of the in-plane field slightly influences the cavity parameters and its flux-dependence, that the sweetspot frequency is slightly varying with magnetic history and that the biasing current is (partly) flowing through the mechanical oscillator. To work around these effects, we automatically bias sweep the cavity prior to each measurement until the desired $\partial\omega_0/\partial\Phi$ is approximately achieved. In most cases, however, the real $\partial\omega_0/\partial\Phi$ slightly deviates from the set value due to the fitting error of ω_0 and due to a non-constant conversion from bias current to flux in particular for higher in-plane fields $B_{||} \sim 10\,\text{mT}$. Typically, this error is around 10% of the set value. To compensate for this, we additionally fit the flux dependence taken during the search for the desired $\partial\omega_0/\partial\Phi$ and extract a more precise number for the real value from there.

Additional data set for g_0 vs $\partial\omega_0/\partial\Phi$

In Supplementary Fig. 11 we show the data from the main paper Fig. 3**e** together with a second data set obtained for $B_{||} = 5\,\text{mT}$, here plotted vs $\partial\omega_0/\partial\Phi$ instead of flux bias to demonstrate the linear dependence more clearly. Both datasets show an approximately linear increase and the ratio between the slopes of the two theoretical lines 2.4 is close to the expected value of 2 arising from the difference in in-plane field. The deviation from the factor 2 seems to be related to a systematic influence of the in-plane field to the system as can also be seen in Fig. 4 of the main paper, where the data points for 5 mT lie under the theoretical line, while the points for 10 mT lie slightly above.

Main paper error bars

The error bars given in the main paper on the data points in Fig. 3**e** and Fig. 4**c** are estimates of ±10% of the extracted value for g_0. The dominant factor for this uncertainty is given by possible variations of the feedline input power with frequency. Typically, we find considerable cable resonances in our setup, cf. Supplementary Fig. 4**a**. Due to our experimental scheme, where the red sideband drive tone is sent to the sample through another cable than the probe tone, cf. Supplementary Fig. 2**a** and **d**, and the drive signal is not going to the amplifier chain, we cannot calibrate for possible cable resonances on the drive tone. We roughly calibrated the input attenuation of the drive line by disconnecting the sample and measuring the reflection of the open connector at base temperature into the amplifier line. The cable resonances, however, might significantly differ, when the sample is connected and thus the frequency dependence is not accessible. This leaves us with possible small uncertainties regarding the intracavity photon number.

Additional sources for errors lie in the fit values of the cavity linewidth, the OMIT amplitude and the mechanical linewidth.

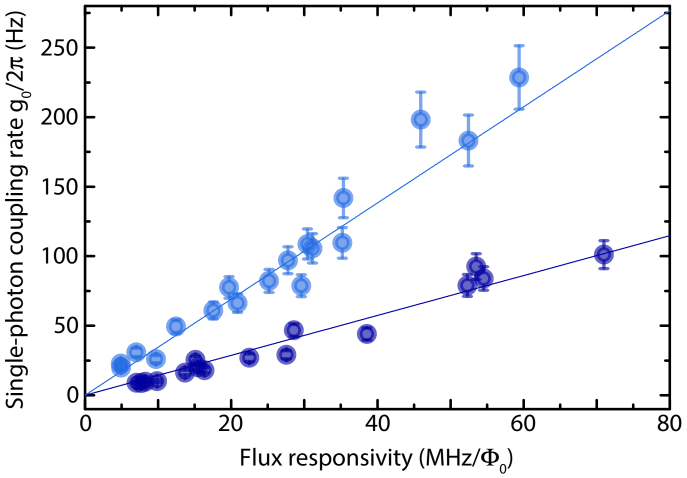

SUPPLEMENTARY FIGURE 11. **Tuning of the single-photon coupling rate g_0 with flux responsivity.** The bright blue points correspond to the values for g_0 extracted for $B_{||} = 10\,\mathrm{mT}$ and the dark points to $B_{||} = 5\,\mathrm{mT}$. The lines correspond to the theoretical calculations, where the theory line for $10\,\mathrm{mT}$ has been scaled by a factor of $1/0.83$.

Accounting for uncertainties in $\partial\omega_0/\partial\Phi$ in the in-plane dependence

In order to see the linear scaling of g_0 with the in-plane magnetic field, the data for different in-plane fields have to be taken for all other conditions fixed, in particular for the flux responsivity $\partial\omega_0/\partial\Phi$ being constant. This biasing procedure is non-trivial in our device due to the metastability of the SQUID, an out-of-plane component of the in-plane field and slightly field-dependent biasing conditions. Therefore, we only give a range for the expected theoretical g_0 in the main paper Fig. 4**c**, assuming possible variations of $\partial\omega_0/\partial\Phi$ of about 10%.

SUPPLEMENTARY REFERENCES

[1] R. Igreja and C. J. Dias, Sensors and Actuators A **112**, 291 (2004)
[2] E. M. Levenson-Falk, R. Vijay, and I. Siddiqi, Applied Physics Letters **98**, 123115 (2011)
[3] R. Vijay, E. M. Levenson-Falk, D. H. Slichter, and I. Siddiqi, Applied Physics Letters **96**, 223112 (2010)
[4] O. W. Kennedy, J. Burnett, J. C. Fenton, N. G. N. Constantino, P. A. Warburton, J. J. L. Morton, and E. Dupont-Ferrier, Physical Review Applied **11**, 014006 (2019).
